# Outcomes of serial sessions of Activa mask combined with intense pulsed light therapy in patients with Meibomian gland dysfunction

**DOI:** 10.1186/s12886-022-02538-0

**Published:** 2022-07-19

**Authors:** Luca Vigo, Marco Pellegrini, Francesco Carones, Vincenzo Scorcia, Giuseppe Giannaccare

**Affiliations:** 1 Carones Ophthalmology Center, Milan, Italy; 2 Department of Translational Medicine, University of Ferrara, Ferrara, Italy; 3 Ospedali Privati Forlì “Villa Igea”, Department of Ophthalmology, Forlì, Italy; 4 Istituto Internazionale per la Ricerca e Formazione in Oftalmologia, Forlì, Italy; 5 Department of Ophthalmology, University “Magna Græcia” of Catanzaro, Viale Europa, 88100 Germaneto (Catanzaro), Italy

**Keywords:** Dry eye, Meibomian gland dysfunction, Active mask, Eye mask, Intense pulsed light, Ocular surface

## Abstract

**Background:**

To evaluate the effects on ocular surface signs and symptoms of serial sessions of heating and vibrating eye mask followed by intense pulsed light (IPL) therapy for the treatment of dry eye disease owing to meibomian gland dysfunction (MGD).

**Methods:**

Consecutive patients with MGD whose signs and symptoms were not satisfactorily controlled with conventional therapy were included. Patients received 3 treatments performed at day 1, 15, and 45 incorporating a session with a newly-developed eye mask (Activa, SBS Sistemi, Turin, Italy) immediately followed by IPL therapy (E > Eye device, E-Swin, Paris, France). Patients were examined before the first session (T0) and 30 days after the last session (T1) for the measurement of: noninvasive break-up time (NIBUT); lipid layer thickness (LLT); tear meniscus height (TMH); meibomian gland loss (MGL); tear osmolarity. Ocular discomfort symptoms were ascertained by ocular surface disease index (OSDI) questionnaire.

**Results:**

Thirty patients were ultimately included in the study. At T1, all objective ocular surface parameters improved significantly, except for TMH: NIBUT and LLT increased from 6.4 ± 1.7 to 8.6 ± 1.7 s and from 57.7 ± 15.5 to 81.3 ± 12.0 μm (all *P* < 0.001), while MGL and tear osmolarity decreased from 21.1 ± 17.3 to 17.0 ± 14.1% and from 302.0 ± 8.5 to 295.7 ± 6.9 mOsm/L (respectively, *P* = 0.004 and *P* < 0.001). In parallel, OSDI score decreased significantly from 49.8 ± 13.5 to 29.8 ± 10.6 (*P* < 0.001). In the historical control group of patients who underwent only IPL, NIBUT, LLT, tear osmolarity and OSDI improved significantly but not MGL and TMH.

**Conclusions:**

Serial sessions incorporating the application of an eye mask producing heating and vibration immediately followed by IPL therapy are able to improve all ocular surface parameters as well as ocular discomfort symptoms in MGD patients.

## Background

Meibomian glands are holocrine sebaceous glands located in the upper and lower eyelids that open on the lid margin where they secrete lipids (meibum) forming the outermost layer of the tear film [1]. The qualitative and quantitative composition of meibum is essential to prevent excessive evaporation of the tear film, and thus to maintain the homeostasis of the ocular surface [2]. In meibomian gland dysfunction (MGD), gland terminal obstruction, occurring due to ductal epithelium keratinization and alteration of the meibum lipids, results in hyposecretion with subsequent tear film instability and increased evaporation [3]. If the disease progresses, acinar degeneration and atrophy may ultimately lead to loss of meibomian glands.

MGD is an area of growing interest since it represents the most common cause of evaporative dry eye disease [4]. Treatment options range from medications such as tear substitutes, essential fatty acids and antibiotics to self-administered eyelid hygiene and warm compresses. In refractory cases, in-office mechanical procedures (meibomian gland expression and probing), heat therapies (e.g. LipiFlow) or light-based treatment (intense pulsed light, IPL) can be employed alone or as complementary therapies to better cope with the disease [5–8].

The goal of MGD treatment is to improve the flow of meibomian gland secretions by liquefying materials that otherwise cause obstruction and by stimulating the function of meibomian glands. Theoretically, this goal could be reached by combining two different procedures in the same session: an eye mask producing heating and vibration could liquefy and release the meibum [9], while IPL could improve the function of meibomian glands through different mechanisms, including ablation of blood vessels, fluidification of meibum, Demodex eradication and reduction of inflammatory cytokines, among others [10–23].

The purpose of the present study was to evaluate the effects on objective parameters of the ocular surface and discomfort symptoms of serial combined treatments incorporating a session of heating and vibrating eye mask followed by IPL therapy.

## Methods

### Study and patients

This prospective pilot study included consecutive patients with MGD who attended for a routine visit the ocular surface office of the University Hospital of Catanzaro (Italy) between February 2021 and September 2021. The study followed the tenets of the Declaration of Helsinki for research involving human subjects. Written informed consent was obtained from all participants after the nature and possible consequences of the study had been explained to them. Patients aged 18–80 years were screened for eligibility according to the following inclusion criteria: presence of MGD defined as the presence of signs consistent with meibomian gland terminal duct obstruction with abnormal quantity and/or quality of meibomian gland secretions; presence of at least one ocular symptom related to MGD (dryness, foreign body sensation, irritation, and burning) not satisfactorily controlled by tear substitutes and eyelid hygiene; pathological value of Ocular Surface Disease Index (OSDI) score (≥ 13); and noninvasive break-up time (NIBUT) < 10 seconds (s) [9]. Patients were excluded from the study if one or more of the following conditions was present: active ocular inflammation; eyelid malposition; recent (within 3 months) ocular surgery; history of contact lens wearing; risk factors for retinal detachment (high myopia, lattice degeneration, and retinal break); and usage of anti-inflammatory eye drops (corticosteroid or cyclosporine) within 1 month [ 9].

### Study treatment

All patients received 3 treatments performed at day 1, 15, and 45 incorporating a session with a newly-developed eye mask (Activa, SBS Sistemi, Turin, Italy) immediately followed by IPL therapy. Briefly, through a fully automated procedure (touch-screen controlled and user- friendly), the mask melts the meibum inside the glands and simultaneously squeezes them. The entire treatment is 15 minutes long, and consists of 2 phases in the following chronological order: 5 minutes of heating at 42 °C (phase I); 10 minutes of combination of heating at 42 °C and vibration up to 20 Hz (phase II) [9]. IPL treatments were performed using E > Eye device (E-Swin, Paris, France), set on the proprietary “dry eye mode”, as previously described [24]. Treatment intensity was determined based on patient’s Fitzpatrick skin type, ranging from 9.8 to 13 J/cm^2^ as per manufacturer recommendations [25]. During each treatment, protective eye shields were placed over the eyes and ultrasound gel was applied to the treatment area. Five flashes were applied for each eye starting from the inner canthus and ending on the temporal region below the lower eyelid, with slight overlapping applications.

### Ocular surface workup

All patients were examined before the first session (T0) and 30 days after the last session (T1) by means of Idra (SBS Sistemi, Turin, Italy) for the automated measurement of the following parameters: NIBUT; lipid layer thickness (LLT); tear meniscus height (TMH); meibomian gland loss (MGL) of upper and lower eyelids [1]. Briefly, NIBUT was measured without the need for fluorescein dye after asking the patient to blink 3 consecutive times and then hold the eyes open. The measurement was repeated 3 times, and the mean value was recorded. LLT was estimated by observing the interference pattern and colours of the moving lipid tear film. TMH was measured along the lower lid margin immediately below the pupil. Infrared meibography was performed in the upper and lower eyelids and MGL was calculated by means of the image editing software ImageJ (National Institute of Health; http://imagej.nih.gov/ij) as the percentage of gland loss in relation to the total tarsal area of the lid. The I-Pen tear osmolarity system (I-Med Pharma Inc., Dollard des-Ormeaux, Quebec, Canada) was used to measure tear osmolarity values. Ocular discomfort symptoms were ascertained by means of OSDI questionnaire.

During the study period, patients were advised to maintain unchanged their ongoing therapy based on tear substitutes instilled 4 times daily and eyelid hygiene before bedtime. Concomitant use of other therapeutic agents (for any ophthalmic disease) was prohibited throughout the study period.

### Sample size

To determine the sample size of the study, an a priori sample size calculation was performed based on the data of the study of Yan et al. [26] In total, the enrollment of 11 patients was required to detect a mean change of 2.3 seconds in TBUT, with a power of 0.95 and a *P* value of 0.05.

### Control group

Data from patients with MGD who have undergone IPL therapy alone at the same time intervals during the study period were retrospectively obtained in order to create an historical control group.

### Statistical analysis

Statistical analysis was conducted using R (version 4.0.0) and RStudio (version 1.2.5042) software. Patients received treatments in both eyes and for statistical analysis data were derived from the eye with the lowest baseline NIBUT value (study eye). The normality of data distribution was assessed by means of the Kolmogorov-Smirnov test. Due to the non-normal distribution of data, the Wilcoxon test was performed to compare ocular surface parameters before and after treatment. A *P* value < 0.05 was considered statistically significant.

## Results

A total of 72 patients with MGD were assessed for eligibility during the study period. Thirty of them (8 males, 22 females; mean age 56.2 ± 13.1 years) fulfilled the criteria and were included in the study. No patient was dropped from the study or used prohibited eye drops, so data from 30 patients were ultimately included in the analysis. Moreover, data from 34 patients (9 males, 25 females; mean age 49.1 ± 12.4 years) who underwent IPL as only therapy in the same period were retrospectively obtained and used as an historical control group. Demographic and clinical characteristics of patients belonging to both groups are reported in Table 1.

**Table 1:** Baseline characteristics of patients enrolled in the study

Parameter	IPL + Activa (study group) (***n*** = 30)	IPL only (control group) (***n*** = 34)
Age (years)	56.2 ± 13.1	49.1 ± 12.4
Gender (M/F)	8/22	9/25
Ethnicity		
European	27 (90%)	30 (88%)
Other	3 (10%)	4 (12%)
Ocular rosacea	6 (20%)	4 (12%)
Ocular allergy	3 (10%)	3 (9%)
Duration of MGD (years)	4.4 ± 2.5	5.1 ± 3.1
History of MGX	15 (50%)	20 (59%)
History of MGP	6 (20%)	6 (18%)

*M* male*, F* female*, MGD* meibomian gland dysfunction*, MGX* meibomian gland expression*, MGP* meibomian gland probing

In patients undergoing Activa mask plus IPL therapy (study group), all objective ocular surface parameters except for TMH improved significantly at T1 compared to T0. In particular, mean values of NIBUT and LLT increased from 6.4 ± 1.7 to 8.6 ± 1.7 s and from 57.7 ± 15.5 to 81.3 ± 12.0 μm (all *P* < 0.001), while mean values of MGL and tear osmolarity decreased from 21.1 ± 17.3 to 17.0 ± 14.1% and from 302.0 ± 8.5 to 295.7 ± 6.9 mOsm/L (respectively, *P* = 0.004 and *P* < 0.001). In parallel, also ocular discomfort symptoms improved and mean OSDI score decreased significantly from 49.8 ± 13.5 at T0 to 29.8 ± 10.6 at T1 (*P* < 0.001). Conversely, mean values of TMH did not change significantly after treatment (*P* = 0.859).

In patients who underwent IPL therapy alone (historical control group), all objective ocular surface parameters except for TMH and MGL improved significantly at T1 compared to T0. In particular, mean values of NIBUT and LLT increased from 7.9 ± 2.9 to 11.4 ± 3.3 s and from 55.3 ± 11.2 to 76.2 ± 15.4 μm, while mean values of tear osmolarity decreased from 304.8 ± 11.1 to 302.0 ± 7.6 mOsm/L; in parallel, mean OSDI score significantly decreased from 44.8 ± 15.5 at T0 to 36.9 to 11.7 at T1 (always *P <* 0.001). Conversely, mean values of TMH and MGL did not change significantly (respectively, *P* = 0.510 and *P* = 0.637). Complete data concerning changes of ocular surface parameters before and after IPL with or without Activa mask are reported in Table 2.

**Table 2:** Ocular surface parameters before and after treatment(s) in the study group and in the control one

Parameter	IPL+Activa (***n***=30)			IPL (control group) (***n***=34)		
	T0	T1	***P***	T0	T1	***P***
**OSDI**	49.8 ± 13.5	29.8 ± 10.6	**< 0.001**	44.8 ± 15.5	36.9 ± 11.7	**< 0.001**
**NIBUT (s)**	6.4 ± 1.7	8.6 ± 1.7	**< 0.001**	7.9 ± 2.9	11.4 ± 3.3	**< 0.001**
**LLT (**μm**)**	57.7 ± 15.5	81.3 ± 12.0	**< 0.001**	55.3 ± 11.2	76.2 ± 15.4	**< 0.001**
**TMH (mm)**	0.24 ± 0.06	0.024 ± 0.04	0.859	0.24 ± 0.05	0.26 ± 0.04	0.510
**MGL (%)**	21.1 ± 17.3	17.0 ± 14.1	**0.004**	28.5 ± 19.3	28.2 ± 17.3	0.637
**Osmolarity (mOsm/L)**	302.0 ± 8.5	295.7 ± 6.9	**< 0.001**	304.8 ± 11.1	302.0 ± 7.6	**< 0.001**

*OSDI* ocular surface disease index*, NIBUT* non-invasive break-up time*, LLT* lipid layer thickness*, TMH* tear meniscus height*, MGL meibomian gland loss*

## Discussion

In the present study, we analyzed the effects on ocular surface in terms of objective signs and subjective symptoms of serial sessions of Activa mask combined with IPL therapy in patients with MGD not responding to conventional therapy. One month after the third session, all objective signs (NIBUT, LLT, MGL and tear osmolarity) improved significantly, except for TMH whose values were within the normal range already at baseline. In parallel, also ocular discomfort symptoms ameliorated significantly after these serial sessions. Further positive results are related to the high compliance of patients who completed the entire session of 3 treatments and avoided the use of prohibited eye drops in the totality of cases throughout the entire study. Positive results were found for the same parameters also in the historical control group that included patients who received IPL as only treatment; however, unlike study group, MGL did not improve significantly in these patients.

The rational of combining an eye mask producing heating and vibration with IPL treatment in patients with MGD originates from the specific function of each procedure: the former is able to reach a therapeutic level of heat that liquefies meibum (phase I) that is then released thanks to the mechanical force exerted on the eyelid by the vibrating function (phase II) [9]; the latter improves the function of meibomian glands through different mechanisms previously demonstrated, such as ablation of blood vessels, fluidification of meibum, Demodex eradication and reduction of inflammatory cytokines [10–23].

Various devices specifically designed for in-office MGD management able to produce heating, heating plus humidity, or heating and massaging have been developed and are currently available in the market [27]. The Activa mask employed in this study was recently validated and preliminary data have shown positive short-term effects (30 minutes after the session) on NIBUT, LLT and ocular discomfort symptoms [9].

IPL is a quite popular procedure employed in the setting of MGD. Despite several studies reported improvement of different parameters, methodological limitations hampered to reach a good level of evidence, and a recent Cochrane review failed to provide a definitive answer regarding its effectiveness and safety [28]. IPL can be used alone or as a helpful supplementary therapy combined with other in-office procedures, especially in recalcitrant MGD cases. Various papers reported positive results obtained by the combined use of IPL with meibomian gland expression [26], meibomian gland probing [29], and low-level light therapy [30].

In the present study, IPL therapy was combined for the first time with Activa mask for 3 in-office sessions. This treatment provided significant improvement of all clinically meaningful metrics in patients with MGD. These positive results highlight the potential synergistic role of these two procedures when combined in the same session. However, beyond the strengths we recognize the limitation of the study design that included an historical control group of patients treated with IPL as the only therapy. A prospective randomized controlled study comparing the techniques used alone or in combination should be conducted in order to confirm and validate these findings.

## Conclusions

In conclusion, the interest and the use of new physical in-office therapies in the setting of MGD are continuously growing. These procedures can be used alone or in combination with other therapies (medical and/or physical) [31]. The present study showed that serial sessions incorporating the application of an eye mask producing heating and vibration immediately followed by IPL therapy are able to improve all ocular surface parameters as well as ocular discomfort symptoms in patients with MGD, while avoiding the need for using other medications (e.g. corticosteroids) apart from tear substitutes.

## Data Availability

The datasets generated and analyzed during the current study are not publicly available due to their proprietary nature, but are available from the corresponding author on reasonable request.
